# Effect of multimorbidity on hypertension management

**DOI:** 10.1038/s41598-023-44813-0

**Published:** 2023-10-31

**Authors:** Eunjeong Ji, Soyeon Ahn, Jung-Yeon Choi, Cheol-Ho Kim, Kwang-il Kim

**Affiliations:** 1 Medical Research Collaborating Center, Seoul National University Bundang Hospital, Seongnam, Republic of Korea; 2 Department of Internal Medicine, Seoul National University Bundang Hospital, Seongnam, Republic of Korea; 3 Department of Internal Medicine, Seoul National University College of Medicine, Seoul, Republic of Korea

**Keywords:** Cardiology, Diseases, Risk factors

## Abstract

Multimorbidity, the coexistence of multiple health conditions, is associated with functional decline, disability, and mortality. We aimed to investigate the effects of multimorbidity on hypertension treatment and control rates by analyzing data from the Korean National Health and Nutrition Examination Survey database, which is a cross-sectional, nationally representative survey conducted by the Korean government. Multimorbidity, defined as having two or more chronic diseases, was evaluated by blood pressure measurements, blood chemistry examinations, and questionnaires. We classified the participants according to the number of multimorbidities from 0 to ≥ 6. Association analysis was performed to identify the patterns of multimorbidity related to hypertension control. From 2016 to 2020, 30,271 adults (≥ 20 years) were included in the analysis (age: 52.1 ± 16.8 years, male: 44.0%), and 14,278 (47.2%) had multimorbidity. The number of chronic conditions was significantly higher in older adults, women, and hypertensive patients. Multimorbidity was associated with hypertension treatment. The number of chronic conditions was significantly higher in controlled compared to uncontrolled patients (3.6 ± 1.7 vs 2.9 ± 1.6, p < 0.001). But the control rate of hypertension among treated patients was lower in patients with multimorbidity (75.6% in hypertension only group vs 71.8% in multimorbidity group, p = 0.009). Multimorbidity patterns showed distinct features in treated and controlled hypertensive patients. In conclusion, multimorbidity has a beneficial effect on the treatment of hypertension, but the control rate of systolic blood pressure was lower among the patients with multimorbidity. More attention should be paid to the hypertensive patients with multimorbidity to improve the control rate of hypertension.

## Introduction

Multimorbidity, defined as the coexistence of two or more chronic diseases in the same individual, is an emerging issue owing to an increase in population aging in developed countries^1,2^. Multimorbidity focuses on the characteristic clustering or patterns of multiple diseases in a single patient and the interactions between diseases and patients. Multimorbidity is more common in older adults than in younger individuals. The prevalence of multimorbidity is over 50% in the elderly, and certain clusters can be found for individual diseases in patients with multimorbidities^3^. Multimorbidity in older patients requires intensive diagnostic workups and is associated with unexpected in-hospital events. In addition, multimorbidity is significantly associated with higher mortality, increased disability, decline in functional status, and lower quality of life (QoL). Consequently, multimorbidity is the primary contributor to increased medical costs, leading to increased healthcare utilization^4–6^.

Hypertension is a common chronic condition associated with an increased risk of cardiovascular disease, disability, and impaired QoL. It frequently manifests with other chronic conditions such as diabetes mellitus, obesity, and depression. Accordingly, it is important to consider multimorbidity in the diagnosis and treatment of patients with hypertension^7,8^. However, data on the effects of multimorbidity on the treatment or control of hypertension are limited. In addition, current guidelines do not sufficiently address older patients with multimorbidity, who are commonly excluded from or underrepresented in major clinical trials; hence, there is little evidence on how to manage elevated blood pressure (BP) in hypertensive patients with multimorbidity.

Multimorbidity shows different patterns, and understanding these patterns is helpful in determining the optimal treatment of certain diseases. Thus, it is important to understand whether certain comorbidities can affect BP over time, which could justify individualized recommendations for BP treatment in such patients^9^.

For this purpose, we analyzed data from the Korean National Health and Nutrition Examination Survey (KNHANES). We aimed to identify the association between multimorbidity and hypertension prevalence, treatment, and control rate, and to investigate the characteristic patterns of multimorbidity related to hypertension treatment or control among KNHANES participants.

## Methods

### Study population

Data were derived from the KNHANES. The KNHANES is a cross-sectional and nationally representative survey conducted by the Division of Chronic Disease Surveillance of the Korea Disease Control and Prevention Agency^10^. KNHANES has been conducted periodically since 1998 to assess the health and nutritional status of community-dwelling Koreans. The survey consists of a health interview survey, nutrition survey, and health examination survey. A stratified multistage probability sampling design was used to select household units.

Among the 31,811 participants older than 20 years who participated in the survey between 2016 and 2020, 1540 were excluded owing to a lack of data regarding BP or hypertension treatment. A total of 30,271 participants, including 13,334 males (44.0%) were included in the analysis. All study procedures were performed in accordance with relevant guidelines and regulations. All surveys were conducted with the written consent of the participants. This study was approved by the Institutional Research Committee of the Seoul National University Bundang Hospital (IRB No. X-2208-773-902).

### Measurement

Trained nurses measured BP using a mercury sphygmomanometer (Baumanometer Desk model 0320, W. A. BAUM, Copiague, NY, USA) with an appropriately sized cuff after the participants sat for at least 5 min to stabilize BP. During the measurement period, the participants were seated, leaning against the back of a chair, with their feet flat on the floor. The participants’ right arm was positioned such that the middle of the cuff was at the heart level. Three BP readings were obtained from each participant after 5 min of rest. The mean of the second and third measured BP values were used in the analysis.

Blood samples were obtained in the morning after at least 8 h of fasting. Blood samples were centrifuged, refrigerated at the examination site, and transferred to a central laboratory in ice boxes. Body weight and height was measured by trained medical staff at the mobile examination center^11^. Body mass index (BMI) was calculated as weight in kilograms divided by height in meters squared.

### Definition of multimorbidity

Among the many chronic conditions, we selected 26 conditions base on previous reports and availability of data in KNHANE^12^. Hypertension was defined as a BP ≥ 140/90 mmHg or current treatment with antihypertensive medication. Hypertension control was defined as having an average systolic and diastolic BP of < 140/90 mmHg. Control rate was defined as the proportion of participants with adequate BP control among those with hypertension or taking antihypertension medication. Hypertension treatment was defined if the participants were taking antihypertensive medications for ≥ 20 days/month.

Hypercholesterolemia was defined as cholesterol ≥ 240 mg/dL or current treatment with cholesterol-lowering agents. Diabetes mellitus was defined as a fasting glucose ≥ 126 mg/dL or treatment with an oral hypoglycemic agent or insulin. Obesity was defined as a BMI ≥ 25 kg/m^2^^13^. Anemia was defined as a hemoglobin level < 13 g/dL in men and < 12 g/dL in women. Chronic kidney disease (CKD) was defined as estimated glomerular filtration rate (eGFR) < 60 mL/min/1.73 m^2^, which was measured using the chronic kidney disease-epidemiology collaboration (CKD-EPI) equation^14^. Hepatitis B infection was determined based on the serum hepatitis B viral surface antigen status using an electrochemiluminescence immunoassay. Hepatitis C infection was determined by anti-HCV antibody testing using a chemiluminescent microparticle immunoassay.

The presence of other diseases was assessed during the health interview, and participants responded to the following question: “Has a doctor ever told you that you have (or have had) stroke, ischemic heart disease, depression, osteoporosis, bronchial asthma, atopy, chronic sinusitis, liver cirrhosis, thyroid disease, rheumatoid or degenerative arthritis, or cancer (lung, stomach, liver, colon, breast, cervix, thyroid, or other)?”.

Among the 26 conditions, we calculated the presence of conditions and classified the participants according to the number of multimorbidities from 0 to ≥ 6.

### Statistical analysis

Statistical analyses were performed using SAS 9.4 for Windows (SAS Institute, Inc., Cary, NC, USA) using the survey procedure and R (version 4.1.1; R Foundation for Statistical Computing, Vienna, Austria). To calculate the total population represented by the sample, stratified variables and sample weights designed by the Korean Centers for Disease Control and Prevention were used. All estimates were weighted, and the sample weights accounted for the unequal probabilities of selection resulting from the complex sample design, survey non-response, and planned oversampling of the selected population subgroups. Continuous variables were expressed as mean ± standard deviation and compared using the unpaired Student’s t-test. Qualitative variables were expressed as counts and percentages, and the Chi-square test was used to compare proportions. Uni- and multivariable logistic models were used to identify the factors affecting the hypertension control. We used association rule analysis to display the pattern of multimorbidity based on R packages “arules” and “arulesViz”. Multimorbidity patterns were visualized as pathways represented by nodes and edges. The size and color of the nodes represent the support and lift, respectively. All statistical analyses were two-tailed, and p-values < 0.05 were considered statistically significant.

## Results

### Baseline characteristics according to the multimorbidity status

The mean age of the study population was 52.1 ± 16.8 years, and men represented 44.0% of the sample. The mean number of chronic diseases was 1.8 ± 1.7 (range 0–15).

Among the 26 chronic conditions, obesity was the most common (35.1%), followed by hypertension (33.2%), hypercholesterolemia (22.7%), diabetes mellitus (12.7%), and arthritis (11.5%).

**Figure 1:**
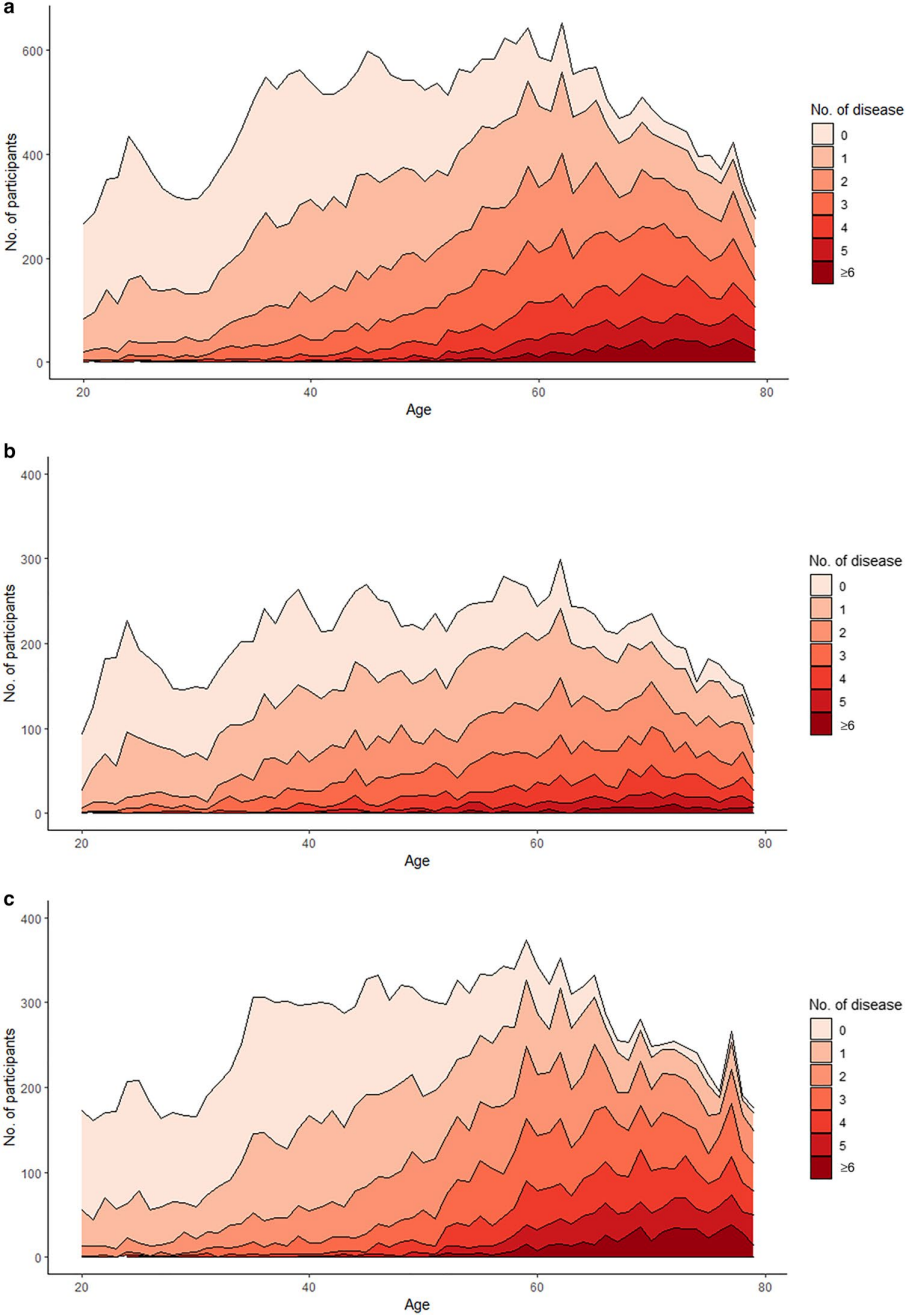
Distribution of the number of chronic medical conditions according to age of participants. Female participants had more chronic diseases than male participants. The mean number of chronic diseases among women was 1.9 ± 1.9, which was significantly greater than that among men (1.7 ± 1.6, p < 0.0001). (**a**) All participants; (**b**) male participants; and (**c**) female participants.

The baseline clinical and laboratory characteristics according to the number of multimorbidities are presented in Table 1. Significant differences were identified in demographic and laboratory characteristics according to the number of multimorbidities. Participants with more multimorbidities were older, female, less active, and had higher BMI, BP, and liver enzymes, and lower eGFR. The prevalence of multimorbidity increased significantly as the participants became older, especially in female participants (Fig. 1).

**Table 1:** Clinical and laboratory characteristics of KNHANES participants according to burden of multimorbidity (N = 30,271).

	Total (N = 30,271)	Multimorbidity = 0 (N = 8444)	Multimorbidity = 1 (N = 7549)	Multimorbidity = 2 (N = 5434)	Multimorbidity = 3 (N = 3748)	Multimorbidity = 4 (N = 2522)	Multimorbidity = 5 (N = 1449)	Multimorbidity ≥ 6 (N = 1125)	p value
Age (year)	52.11 (16.75)	41.87 (14.36)	48.61 (16.04)	55.46 (15.32)	59.86 (13.96)	62.79 (12.55)	65.31 (11.17)	69.35 (8.86)	0.004
Age group, n (%)									
20–39	8009 (26.46)	4081 (48.33)	2428 (32.16)	952 (17.52)	362 (9.66)	142 (5.63)	36 (2.48)	8 (0.71)	< 0.0001
40–59	11,199 (37.00)	3289 (38.95)	3151 (41.74)	2184 (40.19)	1316 (35.11)	764 (30.29)	359 (24.78)	136 (12.09)	
≥ 60	11,063 (36.55)	1074 (12.72)	1970 (26.10)	2298 (42.29)	2070 (55.23)	1616 (64.08)	1054 (72.74)	981 (87.20)	
Male sex, n (%)	13,334 (44.05)	3527 (41.77)	3762 (49.83)	2562 (47.15)	1658 (44.24)	1019 (40.40)	520 (35.89)	286 (25.42)	< 0.0001
House income, n (%)									
1	5070 (16.82)	665 (7.91)	954 (12.69)	1002 (18.50)	821 (22.01)	640 (25.50)	490 (33.89)	498 (44.46)	< 0.0001
2	5606 (18.60)	1246 (14.82)	1316 (17.50)	1099 (20.29)	770 (20.64)	581 (23.15)	344 (23.79)	250 (22.32)	
3	6103 (20.25)	1858 (22.11)	1581 (21.03)	1059 (19.55)	758 (20.32)	450 (17.93)	245 (16.94)	152 (13.57)	
4	6529 (21.66)	2171 (25.83)	1782 (23.70)	1123 (20.73)	737 (19.76)	406 (16.18)	183 (12.66)	127 (11.34)	
5	6837 (22.68)	2465 (29.33)	1885 (25.07)	1133 (20.92)	644 (17.27)	433 (17.25)	184 (12.72)	93 (8.30)	
Marital status, n (%)									
Married	20,838 (68.87)	5421 (64.25)	5349 (70.89)	3975 (73.20)	2724 (72.68)	1779 (70.57)	961 (66.37)	629 (55.91)	0.2239
Single*	9417 (31.13)	3016 (35.75)	2197 (29.11)	1455 (26.80)	1024 (27.32)	742 (29.43)	487 (33.63)	496 (44.09)	
Current smoker, n (%)	5302 (17.73)	1629 (19.50)	1498 (20.14)	946 (17.65)	593 (16.01)	363 (14.54)	175 (12.20)	98 (8.77)	< 0.0001
Alcohol drinking, n (%)	15,907 (53.16)	5179 (61.96)	4370 (58.74)	2783 (51.82)	1675 (45.22)	1076 (43.04)	530 (36.96)	294 (26.30)	< 0.0001
Regular exercise, n (%)	12,150 (42.65)	3815 (47.98)	3232 (45.86)	2098 (41.14)	1392 (39.62)	861 (36.16)	454 (32.59)	298 (27.02)	< 0.0001
BMI (kg/m^2^)	24.02 (3.59)	21.65 (2.08)	24.23 (3.62)	24.81 (3.67)	25.09 (3.52)	25.72 (3.49)	26.21 (3.43)	26.43 (3.29)	0.002
SBP (mmHg)	119.42 (16.71)	109.81 (11.06)	117.74 (15.37)	123.41 (17.43)	125.56 (16.43)	128.36 (16.98)	129.48 (16.84)	130.09 (17.02)	0.002
DBP (mmHg)	75.49 (10.09)	72.53 (7.74)	76.02 (9.68)	77.70 (11.33)	77.05 (10.66)	77.27 (11.26)	76.17 (11.13)	73.25 (10.28)	1
DM, n (%)	3839 (12.68)	0 (0)	314 (4.16)	621 (11.43)	799 (21.32)	736 (29.18)	690 (47.62)	679 (60.36)	< 0.0001
Hypercholesterolemia, n (%)	6865 (24.11)	0 (0)	0 (0)	1153 (22.91)	1832 (51.37)	1736 (71.32)	1133 (80.58)	1011 (91.08)	< 0.0001
BUN (mg/dL)	15.04 (4.87)	13.60 (3.82)	14.40 (4.11)	15.21 (4.47)	16.14 (5.30)	16.75 (5.81)	17.24 (5.74)	18.61 (7.49)	0.002
Creatinine (mg/dL)	0.82 (0.26)	0.79 (0.16)	0.80 (0.17)	0.81 (0.22)	0.84 (0.37)	0.85 (0.33)	0.87 (0.32)	0.94 (0.59)	0.002
WBC (× 10^3^/µL)	6.22 (1.76)	5.99 (1.59)	6.23 (1.76)	6.27 (1.84)	6.30 (1.84)	6.40 (1.79)	6.50 (1.86)	6.50 (1.97)	0.002
GFR (mL/min/1.73 m^2^)	94.66 (18.75)	103.28 (15.19)	98.81 (15.99)	93.11 (16.87)	88.09 (18.02)	84.62 (18.31)	81.09 (20.55)	73.13 (21.94)	0.004
Anemia, n (%)	2847 (9.72)	0 (0)	845 (11.64)	589 (11.31)	460 (12.59)	332 (13.38)	263 (18.47)	358 (32.17)	< 0.0001
Platelet (× 10^3^/µL)	258.31 (63.58)	258.34 (56.75)	261.75 (62.72)	257.93 (64.64)	257.56 (72.10)	255.78 (64.97)	253.38 (68.68)	251.94 (70.17)	0.004
AST (IU/L)	23.84 (15.05)	20.74 (11.3)	23.68 (17.22)	24.94 (13.53)	25.66 (12.21)	26.83 (23.28)	26.81 (14.17)	25.84 (12.48)	0.024
ALT (IU/L)	22.69 (18.45)	17.56 (12.03)	23.25 (19.17)	24.96 (19.92)	25.31 (17.82)	26.99 (25.41)	26.61 (23.27)	22.87 (13.17)	0.264

*BMI* body mass index, *SBP* systolic blood pressure, *DBP* diastolic blood pressure, *DM* diabetes mellitus, *BUN* blood urea nitrogen, *WBC* white blood cell, *GFR* glomerular filtration rate, *AST* aspartate aminotransferase, *ALT* alanine aminotransferase.

Data are presented as number (%) or mean (SD), Trend test was performed, and p values calculated by Jonckheere–Terpstra test for continuous variables and by linear-by-linear test for discrete variables are presented.

*Single means divorced, separated, widowed, or never married.

### Comparison between hypertensive and normotensive patients

Compared with normotensive participants, hypertensive patients were older, male, obese, and less active. In addition, hypertensive patients had more chronic conditions; thus, the number of chronic conditions were significantly higher among the hypertensive patients (3.2 ± 1.7 for the hypertensive patients and 1.1 ± 1.3 for the normotensive patients, p < 0.001) (Table 2).

**Table 2:** Comparison of clinical and laboratory characteristics in normotensive and hypertensive participants (N = 30,271).

	Normotensive (N = 20,207)	Hypertensive (N = 10,064)	p value
Age (year)	46.65 (15.63)	63.05 (13.18)	< 0.0001
Age group, n (%)			
20–39	7403 (36.64)	606 (6.02)	< 0.0001
40–59	8197 (40.57)	3002 (29.83)	
≥ 60	4607 (22.80)	6456 (64.15)	
Male sex, n (%)	8486 (42.00)	4848 (48.17)	< 0.0001
House income, n (%)			
1	2286 (11.35)	2784 (27.81)	< 0.0001
2	3415 (16.96)	2191 (21.89)	
3	4300 (21.36)	1803 (18.01)	
4	4902 (24.35)	1627 (16.25)	
5	5231 (25.98)	1606 (16.04)	
Marital status, n (%)			
Married	13,810 (68.38)	7028 (69.86)	0.0089
Single*	6385 (31.62)	3032 (30.14)	
Current smoker, n (%)	3674 (18.36)	1628 (16.46)	< 0.0001
Alcohol drinking, n (%)	11,189 (55.89)	4718 (47.63)	< 0.0001
Regular exercise, n (%)	8839 (46.13)	3311 (35.50)	< 0.0001
BMI (kg/m^2^)	23.43 (3.43)	25.22 (3.61)	< 0.0001
SBP (mmHg)	112.45 (11.56)	133.41 (16.67)	< 0.0001
DBP (mmHg)	73.41 (7.92)	79.66 (12.42)	< 0.0001
DM, n (%)	1424 (7.05)	2415 (24.00)	< 0.0001
Hypercholesterolemia, n (%)	3277 (17.12)	3588 (38.42)	< 0.0001
Hypertriglyceridemia, n (%)	1865 (11.86)	1459 (18.50)	< 0.0001
BUN (mg/dL)	14.26 (4.21)	16.61 (5.66)	< 0.0001
Creatinine (mg/dL)	0.79 (0.21)	0.86 (0.34)	< 0.0001
WBC (× 10^3^/µL)	6.12 (1.73)	6.42 (1.82)	< 0.0001
GFR (mL/min/1.73 m^2^)	99.49 (16.78)	84.82 (18.72)	< 0.0001
Anemia, n (%)	1723 (8.77)	1124 (11.66)	< 0.0001
Platelet (× 10^3^/µL)	260.44 (61.81)	253.99 (66.82)	< 0.0001
AST (IU/L)	22.70 (13.55)	26.15 (17.48)	< 0.0001
ALT (IU/L)	21.52 (18.25)	25.09 (18.63)	< 0.0001
Number of chronic conditions	1.08 (1.25)	3.23 (1.69)	< 0.0001
Number of chronic conditions, n (%)			
0	8444 (41.79)	0 (0)	< 0.0001
1	6004 (29.71)	1545 (15.35)	
2	2990 (14.80)	2444 (24.28)	
3	1722 (8.52)	2026 (20.13)	
4	690 (3.41)	1832 (18.20)	
5	250 (1.24)	1199 (11.91)	
≥ 6	107 (0.53)	1018 (10.12)	

Data are presented as mean (SD) or number (%).

*BMI* body mass index, *SBP* systolic blood pressure, *DBP* diastolic blood pressure, *DM* diabetes mellitus, *BUN* blood urea nitrogen, *WBC* white blood cell, *GFR* glomerular filtration rate, *AST* aspartate aminotransferase, *ALT* alanine aminotransferase.

*Single means divorced, separated, widowed, or never married.

### Effect of multimorbidity on the prevalence, treatment, and control of hypertension

**Figure 2:**
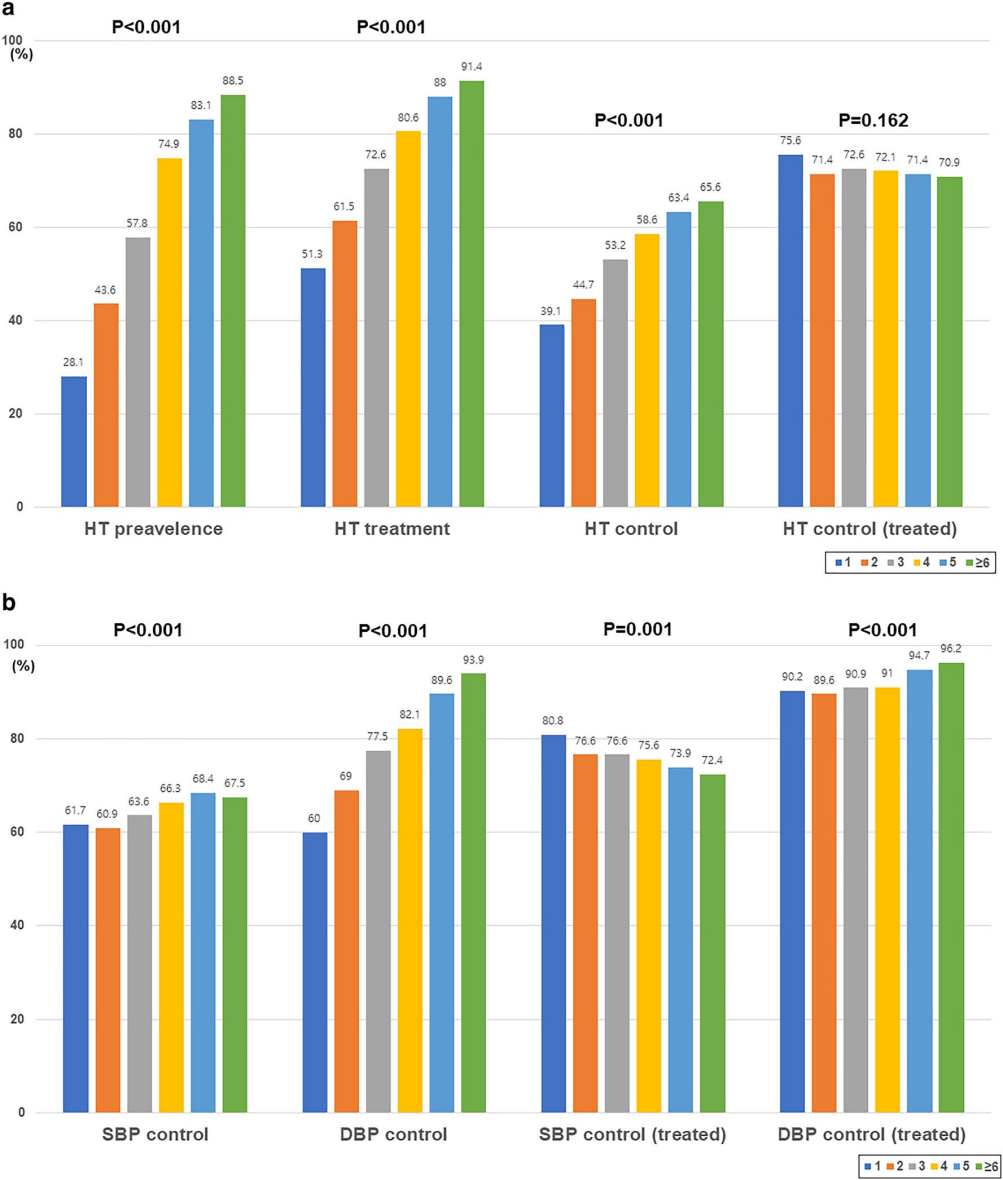
Hypertension prevalence, treatment, and control rate according to multimorbidity. Hypertension prevalence, treatment, and control rates significantly increased with an increase in the burden of multimorbidity. However, the hypertension control rate among the treated patients did not differ according to multimorbidity status. Systolic and diastolic blood pressure control rates were higher in patients with multimorbidities. However, the systolic blood pressure control rate among the treated patients was inversely related to the burden of multimorbidity. (**a**) Hypertension prevalence, treatment, and control rate. (**b**) Systolic and diastolic blood pressure control rates according to the number of multimorbidities.

The prevalence and treatment of hypertension were significantly higher among participants with multimorbidity. In addition, the hypertension control rate was significantly higher in patients with multimorbidity. However, the hypertension control rate among treated hypertensive patients was not significantly different according to the number of chronic conditions (Fig. 2a). Specifically, the control rates of systolic and diastolic BPs were significantly higher among hypertensive participants with multimorbidity. Although the control rate of diastolic BP among treated hypertensive patients was higher in patients with multimorbidity, the control rate of systolic BP was significantly lower in patients with multimorbidity among treated hypertensive patients (Fig. 2b).

### Comparison between uncontrolled and controlled hypertensive patients

**Figure 3:**
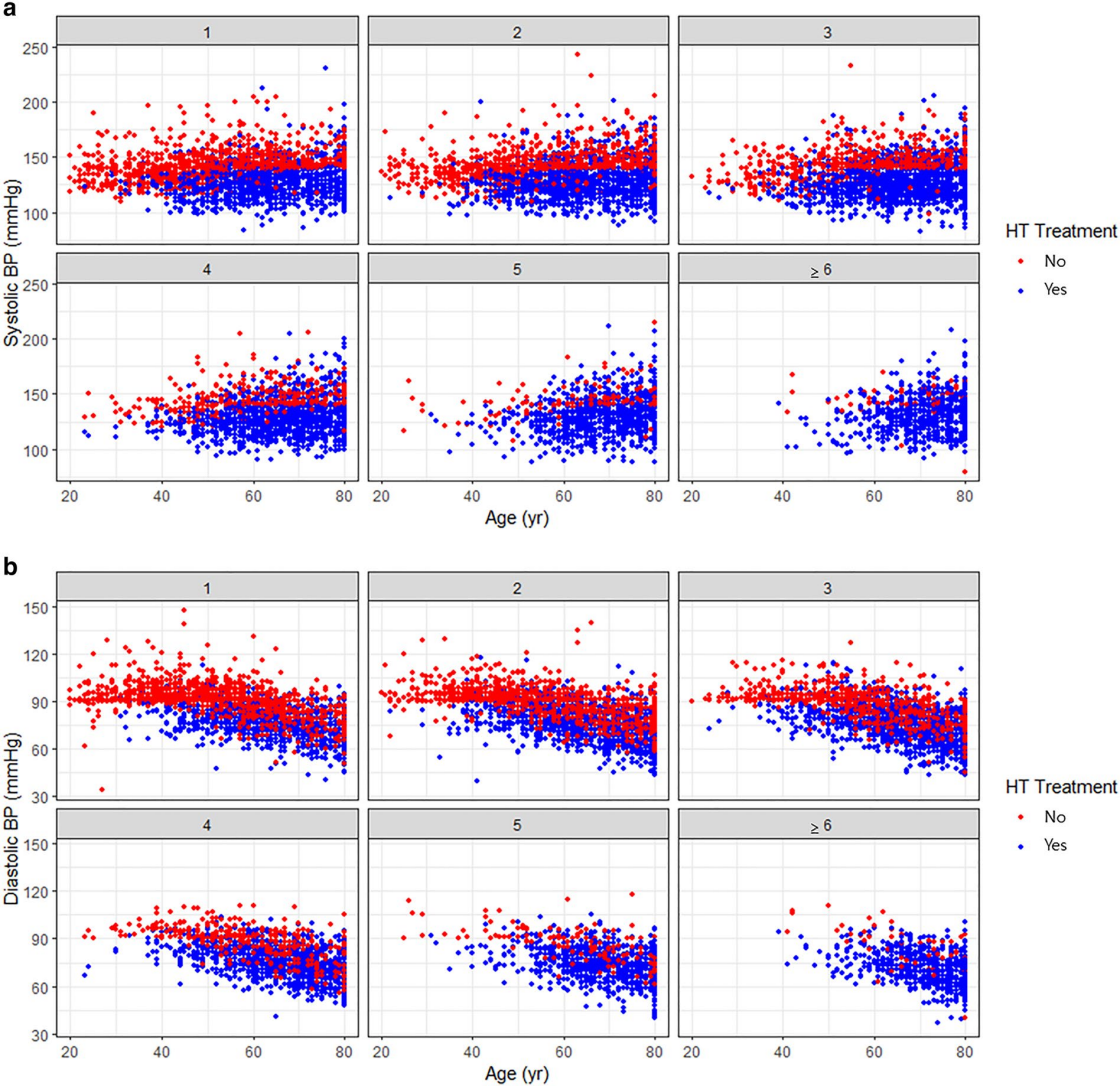
Distribution of blood pressure according to age and hypertension treatment among the hypertensive patients. Most participants with higher blood pressure (≥ 140 mmHg of systolic blood pressure or ≥ 90 mmHg of diastolic blood pressure) were untreated and younger patients among the hypertension only or hypertension plus one other comorbidity group (multimorbidity groups 1 and 2). However, among the multimorbid group (multimorbidity groups 5 and ≥ 6), most of the participants with higher blood pressure were treated patients. (**a**) Systolic blood pressure and (**b**) diastolic blood pressure.

The distributions of systolic and diastolic BPs according to age and hypertension treatment are presented in Fig. 3. Most younger (< 40 years) and untreated patients with hypertension showed higher systolic and diastolic BPs. In contrast, most patients with multimorbidity were treated with antihypertensive medications, but some showed higher systolic and diastolic BPs despite hypertension treatment. Compared to controlled hypertensive patients, uncontrolled hypertensive patients were younger, male, and had an unhealthy lifestyle (alcohol intake, smoking), but had fewer chronic conditions. The number of chronic conditions among the uncontrolled patients was 2.9 ± 1.6, but the number of chronic conditions among the controlled hypertensive patients was 3.6 ± 1.7 (p < 0.001) (Table 3). Although, multimorbidity was more common among female participants, there was no significant gender difference in the relationship between multimorbidity and hypertension control (Supplementary Table 1). In the multivariable analysis, age, sex, marital status, alcohol consumption, diabetes mellitus, hypercholesterolemia, hypertriglyceridemia, and the number of chronic conditions were independent variables associated with hypertension control among hypertensive patients (Table 4).

**Table 3:** Comparison of clinical and laboratory characteristics in controlled and uncontrolled hypertensive patients.

	All hypertensive patients (N = 10,064)			Treated hypertensive patients (N = 6998)		
	Controlled (N = 5121)	Uncontrolled (N = 4943)	p value	Controlled (N = 5070)	Uncontrolled (N = 1928)	p value
Age (year)	66.36 (10.50)	59.63 (14.72)	< 0.0001	66.37 (10.49)	67.21 (10.82)	0.003
Age group, n (%)						
20–39	63 (1.23)	543 (10.99)	< 0.0001	62 (1.22)	23 (1.19)	0.8396
40–59	1213 (23.69)	1789 (36.19)		1198 (23.63)	443 (22.98)	
≥ 60	3845 (75.08)	2611 (52.82)		3810 (75.15)	1462 (75.83)	
Male sex, n (%)	2425 (47.35)	2423 (49.02)	0.0947	2403 (47.40)	745 (38.64)	< 0.0001
House income, n (%)						
1	1595 (31.29)	1189 (24.20)	< 0.0001	1577 (31.25)	656 (34.26)	0.0081
2	1193 (23.40)	998 (21.65)		1183 (23.44)	438 (22.87)	
3	833 (16.34)	970 (19.74)		823 (16.31)	341 (17.81)	
4	729 (14.30)	898 (18.28)		720 (14.27)	239 (12.48)	
5	748 (14.67)	858 (17.46)		744 (14.74)	241 (12.58)	
Marital status, n (%)						
Married	3623 (70.79)	3405 (68.90)	0.0389	3587 (70.78)	1251 (64.89)	< 0.0001
Single*	1495 (29.21)	1537 (31.10)		1481 (29.22)	677 (35.11)	
Current smoker, n (%)	743 (14.74)	885 (18.24)	< 0.0001	736 (14.76)	215 (11.36)	0.0003
Alcohol drinking, n (%)	2260 (44.81)	2458 (50.57)	< 0.0001	2239 (44.84)	747 (39.38)	< 0.0001
Regular exercise, n (%)	1561 (32.99)	1750 (38.08)	< 0.0001	1547 (33.02)	571 (31.79)	0.3457
BMI (kg/m^2^)	25.25 (3.38)	25.19 (3.84)	0.4465	25.25 (3.38)	25.20 (3.42)	0.5258
SBP (mmHg)	122.04 (10.43)	145.18 (13.45)	< 0.0001	122.03 (10.42)	147.72 (12.42)	< 0.0001
DBP (mmHg)	72.76 (8.90)	86.80 (11.47)	< 0.0001	72.74 (8.88)	81.71 (11.31)	< 0.0001
DM, n (%)	1536 (29.99)	879 (17.78)	< 0.0001	1521 (30.00)	538 (27.90)	0.0857
Hypercholesterolemia, n (%)	2086 (44.34)	1502 (32.41)	< 0.0001	2068 (44.39)	801 (44.70)	0.8216
Hypertriglyceridemia, n (%)	633 (15.66)	826 (21.49)	< 0.0001	626 (15.65)	278 (17.99)	0.0339
BUN (mg/dL)	17.20 (5.67)	16.01 (5.58)	< 0.0001	17.22 (5.68)	17.53 (6.46)	0.0753
Creatinine (mg/dL)	0.87 (0.34)	0.86 (0.35)	0.0122	0.87 (0.34)	0.89 (0.50)	0.2983
WBC (× 10^3^/µL)	6.41 (1.85)	6.42 (1.78)	0.8409	6.42 (1.85)	6.42 (1.79)	0.8632
GFR (mL/min/1.73 m^2^)	81.70 (18.16)	88.04 (18.74)	< 0.0001	81.66 (18.16)	80.21 (19.80)	0.0066
Anemia, n (%)	678 (13.86)	446 (9.39)	< 0.0001	670 (13.83)	260 (14.18)	0.7184
Platelet (× 10^3^/µL)	251.53 (69.09)	256.51 (64.30)	0.0002	251.51 (69.14)	251.02 (65.30)	0.7914
AST (IU/L)	25.81 (18.00)	26.50 (16.91)	0.0506	25.83 (18.08)	25.81 (11.20)	0.9751
ALT (IU/L)	24.01 (16.76)	26.20 (20.32)	< 0.0001	24.03 (16.79)	23.77 (16.06)	0.5613
Number of chronic conditions	3.55 (1.73)	2.91 (1.58)	< 0.0001	3.55 (1.74)	3.59 (1.70)	0.3551
Number of chronic conditions, n (%)						
1	570 (11.13)	975 (19.72)	< 0.0001	566 (11.16)	202 (10.48)	0.8432
2	1037 (20.25)	1407 (28.46)		1026 (20.24)	376 (19.50)	
3	1069 (20.87)	957 (19.36)		1055 (20.81)	402 (20.85)	
4	1035 (20.21)	797 (16.12)		1027 (20.26)	393 (20.38)	
5	726 (14.18)	473 (9.57)		719 (14.18)	293 (15.20)	
≥ 6	684 (13.36)	334 (6.76)		677 (13.35)	262 (13.59)	

Data are presented as mean (SD) or number (%).

*BMI* body mass index, *SBP* systolic blood pressure, *DBP* diastolic blood pressure, *DM* diabetes mellitus, *BUN* blood urea nitrogen, *WBC* white blood cell, *GFR* glomerular filtration rate, *AST* aspartate aminotransferase, *ALT* alanine aminotransferase.

*Single means divorced, separated, widowed, or never married.

**Table 4:** Factor associated with hypertension control among hypertensive patients (N = 10,064).

	Univariable analysis				Multivariable analysis				Multivariable analysis			
	OR	95% CI		p value	OR	95% CI		p value	OR	95% CI		p value
Age (year)	0.956	0.952–0.96		< 0.0001	0.96	0.953–0.966		< 0.0001	0.96	0.953–0.966		< 0.0001
Sex (Female vs male)	0.779	0.711–0.854		< 0.0001	1.169	1.009–1.355		0.0382	1.163	1.003–1.349		0.0452
House income												
2 vs 1	1.196	1.043–1.372		0.0105	0.881	0.741–1.047		0.1486	0.882	0.742–1.048		0.1541
3 vs 1	1.642	1.415–1.906		< 0.0001	1.039	0.854–1.265		0.7009	1.053	0.865–1.281		0.6073
4 vs 1	1.727	1.486–2.007		< 0.0001	0.958	0.781–1.175		0.6799	0.964	0.787–1.182		0.7257
5 vs 1	1.526	1.31–1.778		< 0.0001	0.8	0.646–0.99		0.0399	0.8	0.646–0.99		0.0401
Marital status (Single vs married)	1.207	1.087–1.339		0.0004	1.359	1.176–1.57		< 0.0001	1.357	1.174–1.568		< 0.0001
Current smoker (Smoker vs non-smoker)	1.432	1.266–1.62		< 0.0001	0.952	0.8–1.133		0.5782	0.946	0.794–1.127		0.5314
Alcohol drinking (Yes vs no)	1.332	1.212–1.465		< 0.0001	0.858	0.748–0.986		0.0304	0.852	0.742–0.978		0.0228
Regular exercise (Yes vs no)	1.299	1.176–1.436		< 0.0001	1.039	0.92–1.174		0.5352	1.04	0.921–1.174		0.5262
BMI (kg/m^2^)	1.009	0.996–1.022		0.1804								
DM (Yes vs no)	0.497	0.443–0.558		< 0.0001	0.692	0.59–0.811		< 0.0001	0.69	0.588–0.809		< 0.0001
Hypercholesterolemia (Yes vs no)	0.553	0.497–0.615		< 0.0001	0.797	0.662–0.958		0.0159	0.787	0.65–0.953		0.0141
Hypertriglyceridemia(Yes vs no)	1.585	1.388–1.81		< 0.0001	1.426	1.228–1.655		< 0.0001	1.43	1.231–1.661		< 0.0001
BUN (mg/dL)	0.947	0.936–0.958		< 0.0001	0.99	0.977–1.003		0.1429	0.99	0.977–1.004		0.1609
Creatinine (mg/dL)	0.894	0.764–1.046		0.1625								
WBC (× 10^3^/µL)	1.033	1.007–1.06		0.0133	0.998	0.963–1.034		0.905	0.997	0.962–1.034		0.8823
GFR (mL/min/1.73 m^2^)	1.023	1.02–1.026		< 0.0001	1	0.996–1.005		0.928	1	0.996–1.005		0.856
Anemia (Yes vs no)	0.536	0.461–0.623		< 0.0001	1.057	0.852–1.31		0.6154	1.051	0.85–1.301		0.6434
Platelet (× 10^3^/µL)	1.002	1.001–1.002		< 0.0001	1	0.999–1.001		0.5344	1	0.999–1.001		0.5534
AST (IU/L)	1.004	0.999–1.009		0.0869								
ALT (IU/L)	1.01	1.007–1.013		< 0.0001	1.002	0.998–1.006		0.2652	1.002	0.999–1.006		0.2325
Number of chronic conditions	0.765	0.742–0.788		< 0.0001	0.876	0.822–0.932		< 0.0001				
Number of chronic conditions												
2 vs 1	0.803	0.689–0.936		0.0052					0.741	0.612–0.896		0.002
3 vs 1	0.493	0.418–0.582		< 0.0001					0.576	0.462–0.718		< 0.0001
4 vs 1	0.431	0.366–0.508		< 0.0001					0.571	0.441–0.739		< 0.0001
5 vs 1	0.369	0.306–0.445		< 0.0001					0.564	0.412–0.772		0.0004
6 vs 1	0.218	0.179–0.266		< 0.0001					0.435	0.303–0.624		< 0.0001

*OR* odds ratio, *CI* confidence interval, *BMI* body mass index, *SBP* systolic blood pressure, *DBP* diastolic blood pressure, *DM* diabetes mellitus, *BUN* blood urea nitrogen, *WBC* white blood cell, *GFR* glomerular filtration rate, *AST* aspartate aminotransferase, *ALT* alanine aminotransferase, *Hypertriglyceridemia* triglyceride ≥ 200 mg/dL.

### Multimorbidity pattern according to hypertension control status

**Figure 4:**
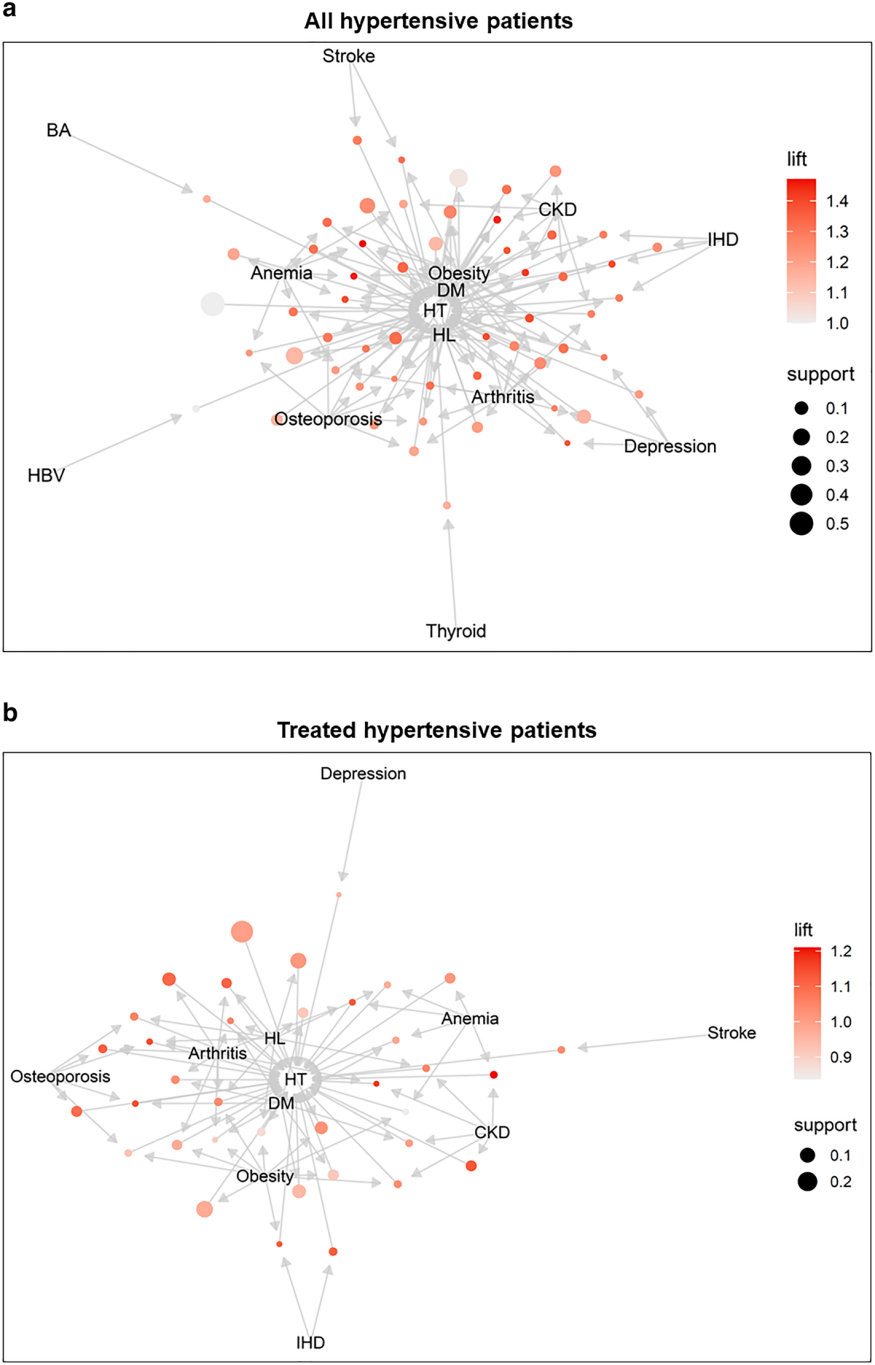
Multimorbidity pattern according to hypertension control among all or treated hypertensive patients. Multimorbidity patterns related to hypertension control among all patients with hypertension or those without hypertension control despite antihypertensive treatments are presented. The balloon size represents support and the color indicates lift. Greater lift values (≫ 1) indicate stronger associations. (**a**) All hypertensive patients, and (**b**) treated hypertensive patients.

The pattern of multimorbidity in controlled hypertensive patients or uncontrolled hypertensive patients despite antihypertensive treatment are shown in Fig. 4. The top 20 case rules by lift are shown in Supplementary Tables 2 and 3. Among patients with hypertension, the highest lift in the association rules for hypertension control was a combination of diabetes mellitus, obesity, and anemia. The highest lift in association rules of the uncontrolled patients among the treated hypertensive patients were CKD and anemia, followed by CKD and arthritis.

## Discussion

We showed that multimorbidity was common among older adults and women. Interestingly, multimorbidity was associated with hypertension treatment, and patients with multimorbidity were more likely to be treated with antihypertensive medications. However, the control rate of systolic BP among the treated patients was lower in those with multimorbidities.

The prevalence of chronic diseases such as hypertension, diabetes mellitus, and dyslipidemia increases with age. Furthermore, complications of chronic medical conditions such as myocardial infarction, stroke, heart failure, chronic kidney disease, and dementia also increase steeply with age. Accordingly, the risk of multimorbidity was significantly higher in older patients. It has been reported that most older patients present with more than two chronic medical conditions^15^, suggesting that multimorbidity is a common problem in older adults.

Multimorbidity has a negative clinical impact; it reduces life expectancy, affects an individual’s QoL and ability to work, increases the risk of hospitalization, and leads to the excessive use of healthcare resources^16^. Previous studies showed the importance of patients-centered care for the patients with multimorbidity^17,18^ However, this study showed that multimorbidity had a beneficial effect on the likelihood of receiving hypertension treatment. Having multiple medical conditions may increase the likelihood of visiting a hospital for disease care. Accordingly, we can assume that multimorbidity has a beneficial effect on the awareness or treatment of hypertension.

However, we observed that the control rate of systolic BP in patients with multimorbidity was lower than that in patients without multimorbidity. The hypertension control rate is reportedly lower among patients with multimorbidities^19^. Drug resistance or impaired organ function may be associated with poor BP control in patients with multimorbidities.

In this study, we showed that age, sex, marital status, and multimorbidity were independently associated with hypertension control in patients with hypertension. Up to two-thirds of patients with hypertension have other comorbidities^20^. However, patients with multimorbidity are commonly excluded from or underrepresented in major clinical trials, thus limiting the evidence on how best to manage elevated BP in patients with hypertension and multimorbidity. With the expected increase in the burden of multimorbidity and hypertension, it is important to understand how the presence of comorbidities affects BP to inform future policies and research. Additionally, multimorbidity is strongly associated with polypharmacy. Thus, it is necessary to consider the interactive effects of drug-drug or drug-disease interactions in patients with hypertension.

The pattern of multimorbidity differed between patients with controlled and uncontrolled hypertension. Moreover, several principal chronic diseases had a larger impact on the control of hypertension. Thus, there is a need to focus on managing key diseases to improve BP control. In this study, we showed that hypertension control was strongly associated with other morbidities such as diabetes, anemia, and CKD.

This study has several limitations. Because we used data from a nationally representative survey, the results can be generalized to the non-institutionalized Korean population. However, these results cannot be applied to other populations such as those from other countries or ethnic groups. Due to the study design, it is impossible to analyse cause and effect between multimorbidities and hypertension control. Finally, patients responded to a survey regarding their comorbidities instead of being tested, so some comorbidities may have gone unreported. Finally, KNHANES dataset do not provide information regarding medication or treatment, which may have an impact on the relationship between multimorbidity and hypertension management.

In conclusion, multimorbidity has a positive effect on the treatment of hypertension; however, the control rate of systolic blood pressure is poor among patients with multiple chronic conditions. More attention should be paid to hypertensive patients with multimorbidities to improve the control rate of hypertension.

### Supplementary Information

Supplementary Tables.

## Data Availability

The dataset used in this study is publicly available online (https://knhanes.kdca.go.kr/knhanes/eng/index.do).
