# Supplementary material for: Effect of multimorbidity on hypertension management
Source: Sci Rep. 2023 Oct 31;13:18764. doi: 10.1038/s41598-023-44813-0 (PMC10618203; doi:10.1038/s41598-023-44813-0)
Supplement: Supplementary file 1 — Supplementary Tables. [file 41598_2023_44813_MOESM1_ESM.docx]

**Supplementary Table 1. Comparison of clinical and laboratory characteristics in controlled and uncontrolled hypertensive patients**

|  |  | **Male** | | | **Female** | | |
| --- | --- | --- | --- | --- | --- | --- | --- |
|  |  | **Controlled (N=2425)** | **Uncontrolled (N=2423)** | **P value** | **Controlled (N=2696)** | **Uncontrolled (N=2520)** | **P value** |
| Age (year) |  | 65.10 (10.79) | 55.11 (15.18) | <.0001 | 67.49 (10.10) | 63.97 (12.84) | <.0001 |
| Age group, n (%) | 20-39 | 40 (1.65) | 418 (17.25) | <.0001 | 23 (0.85) | 125 (4.96) | <.0001 |
|  | 40-59 | 650 (26.80) | 1050 (43.33) |  | 563 (20.88) | 739 (29.33) |  |
|  | ≥60 | 1735 (71.55) | 955 (39.41) |  | 2110 (78.26) | 1656 (65.71) |  |
| House income, n (%) | 1 | 616 (25.51) | 398 (16.51) | <.0001 | 979 (36.49) | 791(31.61) | <.0001 |
|  | 2 | 542 (22.44) | 474 (19.66) |  | 651 (24.26) | 524 (20.94) |  |
|  | 3 | 427 (17.68) | 501 (20.78) |  | 406 (15.13) | 469 (18.75) |  |
|  | 4 | 390 (16.15) | 493 (20.45) |  | 339 (12.64) | 405 (16.19) |  |
|  | 5 | 440 (18.22) | 545 (22.60) |  | 308 (11.48) | 313 (12.51) |  |
| Marital status, n (%) | Married | 2070 (85.36) | 1867 (77.05) | <.0001 | 1553 (57.67) | 1538 (61.06) | 0.0128 |
|  | Single | 355 (14.64) | 556 (22.95) |  | 1140 (42.33) | 981 (38.94) |  |
| Current smoker, n (%) |  | 645 (26.98) | 791 (33.22) | <.0001 | 98 (3.70) | 94 (3.80) | 0.8482 |
| Alcohol drinking, n (%) |  | 1596 (66.67) | 1744 (73.06) | <.0001 | 664 (25.06) | 714 (28.86) | 0.0022 |
| Regular exercise, n (%) |  | 860 (38.00) | 982 (43.53) | 0.0002 | 701 (28.39) | 768 (32.83) | 0.0008 |
| BMI (kg/m^2^) |  | 25.18 (3.23) | 25.51 (3.76) | 0.0009 | 25.31 (3.51) | 24.88 (3.89) | <.0001 |
| SBP (mmHg) |  | 121.48 (10.42) | 142.25 (13.29) | <.0001 | 122.55 (10.41) | 148.00 (13.00) | <.0001 |
| DBP (mmHg) |  | 73.23 (8.98) | 89.47 (11.00) | <.0001 | 72.33 (8.80) | 84.24 (11.33) | <.0001 |
| DM, n (%) |  | 771 (31.79) | 437 (18.04) | <.0001 | 765 (28.38) | 442 (17.54) | <.0001 |
| Hypercholesterolemia, n (%) |  | 895 (39.57) | 579 (25.32) | <.0001 | 1191 (48.75) | 923 (39.33) | <.0001 |
| Hypertriglyceridemia, n (%) |  | 344 (18.11) | 541 (29.64) | <.0001 | 289 (13.49) | 285 (14.12) | 0.5598 |
| BUN (mg/dL) |  | 17.52 (5.85) | 15.79 (5.37) | <.0001 | 16.92 (5.50) | 16.22 (5.78) | <.0001 |
| Creatinine (mg/dL) |  | 1.01 (0.36) | 0.98 (0.35) | 0.0036 | 0.75 (0.26) | 0.74 (0.31) | 0.1221 |
| WBC (x 10^3^/µL) |  | 6.72 (1.89) | 6.76 (1.82) | 0.5069 | 6.13 (1.77) | 6.09 (1.67) | 0.442 |
| GFR (ml/min/1.73 m^2^) |  | 82.09 (17.72) | 90.61 (18.10) | <.0001 | 81.34 (18.56) | 85.54 (19.02) | <.0001 |
| Anemia, n (%) |  | 270 (11.51) | 132 (5.63) | <.0001 | 408 (16.01) | 314 (13.06) | 0.0033 |
| Platelet (x 10^3^/µL) |  | 243.24 (71.85) | 251.80 (62.48) | <.0001 | 259.15 (65.55) | 261.12 (65.72) | 0.2916 |
| AST (IU/L) |  | 26.84 (22.75) | 28.57 (20.19) | 0.006 | 24.85 (12.01) | 24.49 (12.63) | 0.2966 |
| ALT (IU/L) |  | 26.36 (17.49) | 31.20 (23.77) | <.0001 | 21.85 (15.75) | 21.33 (14.72) | 0.2229 |
| Number of chronic conditions |  | 3.20 (1.58) | 2.54 (1.34) | <.0001 | 3.86 (1.81) | 3.27 (1.70) | <.0001 |
| Number of chronic conditions, n (%) | 1 | 336 (13.86) | 575 (23.73) | <.0001 | 234 (8.68) | 400 (15.87) | <.0001 |
|  | 2 | 600 (24.74) | 836 (34.50) |  | 437 (16.21) | 571 (22.66) |  |
|  | 3 | 520 (21.44) | 456 (18.82) |  | 549 (20.36) | 501 (19.88) |  |
|  | 4 | 478 (19.71) | 329 (13.58) |  | 557 (20.66) | 468 (18.57) |  |
|  | 5 | 295 (12.16) | 160 (6.60) |  | 431 (15.99) | 313 (12.42) |  |
|  | ≥6 | 196 (8.08) | 67 (2.77) |  | 488 (18.10) | 267 (10.60) |  |

BMI, body mass index; SBP, systolic blood pressure; DBP, diastolic blood pressure; DM, diabetes mellitus; BUN, blood urea nitrogen; WBC, white blood cell; GFR, glomerular filtration rate; AST, aspartate aminotransferase; ALT, alanine aminotransferase. Data are presented as mean (SD) or number (%)

*; Single means divorced, separated, widowed, or never married

**Supplementary Table 2. Variables associated with hypertension control**

| lhs | support | confidence | coverage | lift |
| --- | --- | --- | --- | --- |
| {DM,Obesity,Anemia} | 0.0127186 | 0.748538 | 0.0169913 | 1.4710577 |
| {Obesity,Anemia,HL} | 0.0121224 | 0.7393939 | 0.0163951 | 1.4530874 |
| {DM,CKD,Obesity} | 0.0153021 | 0.7368421 | 0.0207671 | 1.4480724 |
| {DM,CKD,HL} | 0.0132154 | 0.7228261 | 0.018283 | 1.4205276 |
| {DM,IHD} | 0.0124205 | 0.7102273 | 0.0174881 | 1.3957679 |
| {DM,Anemia,HL} | 0.0120231 | 0.7076023 | 0.0169913 | 1.3906092 |
| {DM,Arthritis,Obesity} | 0.0218601 | 0.7073955 | 0.0309022 | 1.3902028 |
| {DM,Arthritis,Obesity,HL} | 0.0127186 | 0.7071823 | 0.0179849 | 1.3897838 |
| {Depression,Arthritis} | 0.0101351 | 0.6986301 | 0.0145072 | 1.3729767 |
| {CKD,Obesity,HL} | 0.0126192 | 0.6978022 | 0.0180843 | 1.3713496 |
| {DM,Arthritis,HL} | 0.0198728 | 0.6825939 | 0.0291137 | 1.3414615 |
| {DM,CKD} | 0.0273251 | 0.6823821 | 0.0400437 | 1.3410455 |
| {DM,Obesity,HL} | 0.0449126 | 0.6817496 | 0.0658784 | 1.3398024 |
| {CKD,HL} | 0.0247417 | 0.6747967 | 0.0366653 | 1.3261383 |
| {DM,HL} | 0.076411 | 0.6745614 | 0.113275 | 1.3256758 |
| {Obesity,Anemia} | 0.027027 | 0.6732673 | 0.0401431 | 1.3231327 |
| {Stroke,Obesity} | 0.0114269 | 0.6725146 | 0.0169913 | 1.3216534 |
| {Osteoporosis,Arthritis,Obesity} | 0.0183824 | 0.6702899 | 0.0274245 | 1.3172812 |
| {DM,Anemia} | 0.0258347 | 0.6701031 | 0.0385533 | 1.3169142 |
| {DM,Arthritis} | 0.0347774 | 0.6679389 | 0.0520668 | 1.3126611 |

**Supplementary Table 3. Variables associated with uncontrolled hypertension among treated patients**

| lhs | support | confidence | coverage | lift |
| --- | --- | --- | --- | --- |
| {CKD,Anemia} | 0.0151472 | 0.3333333 | 0.0454416 | 1.209889 |
| {CKD,Arthritis} | 0.0102887 | 0.32287 | 0.0318663 | 1.171911 |
| {Osteoporosis,Arthritis,HL} | 0.011146 | 0.3157895 | 0.0352958 | 1.146211 |
| {DM,Osteoporosis} | 0.0112889 | 0.314741 | 0.0358674 | 1.142405 |
| {CKD} | 0.0400114 | 0.3121516 | 0.1281795 | 1.133007 |
| {Arthritis,Anemia} | 0.0117176 | 0.3106061 | 0.0377251 | 1.127397 |
| {IHD,Obesity} | 0.0102887 | 0.3090129 | 0.0332952 | 1.121614 |
| {IHD} | 0.019577 | 0.3085586 | 0.0634467 | 1.119965 |
| {Osteoporosis,Arthritis} | 0.0220063 | 0.308 | 0.071449 | 1.117938 |
| {Arthritis,HL} | 0.0340097 | 0.3059126 | 0.1111746 | 1.110361 |
| {Arthritis} | 0.0721635 | 0.3044002 | 0.2370677 | 1.104872 |
| {Osteoporosis} | 0.0400114 | 0.3013994 | 0.1327522 | 1.09398 |
| {Osteoporosis,HL} | 0.0182909 | 0.2902494 | 0.063018 | 1.053509 |
| {CKD,HL} | 0.0144327 | 0.2893983 | 0.0498714 | 1.05042 |
| {DM,Arthritis,HL} | 0.0115747 | 0.2892857 | 0.0400114 | 1.050011 |
| {Arthritis,Obesity,HL} | 0.0185767 | 0.2863436 | 0.0648757 | 1.039332 |
| {Stroke} | 0.0144327 | 0.2853107 | 0.0505859 | 1.035583 |
| {DM,Arthritis} | 0.0197199 | 0.285124 | 0.0691626 | 1.034905 |
| {CKD,Obesity} | 0.0165762 | 0.2843137 | 0.0583024 | 1.031964 |
| {Obesity,HL} | 0.0620177 | 0.2814527 | 0.2203487 | 1.02158 |
